# Le Cœur en Sabot: shape associations with adverse events in repaired tetralogy of Fallot

**DOI:** 10.1186/s12968-022-00877-x

**Published:** 2022-08-04

**Authors:** Anna Mîra, Pablo Lamata, Kuberan Pushparajah, Georgina Abraham, Charlène A. Mauger, Andrew D. McCulloch, Jeffrey H. Omens, Malenka M. Bissell, Zach Blair, Tyler Huffaker, Animesh Tandon, Sandy Engelhardt, Sven Koehler, Thomas Pickardt, Philipp Beerbaum, Samir Sarikouch, Heiner Latus, Gerald Greil, Alistair A. Young, Tarique Hussain

**Affiliations:** 1 Department of Biomedical Engineering, King’s College London, 1 Lambeth Palace Road, London, SE1 7EU UK; 2 Department of Congenital Heart Disease, Evelina London Children’s Hospital, London, UK; 3 Department of Anatomy and Medical Imaging, University of Auckland, Auckland, New Zealand; 4 Department of Bioengineering, University of California San Diego, San Diego, CA USA; 5 Department of Medicine, University of California San Diego, San Diego, CA USA; 6 Leeds Institute of Cardiovascular and Metabolic Medicine, University of Leeds, Leeds, England; 7 Department of Pediatrics, Division of Pediatric Cardiology, University of Texas Southwestern Medical Center, Dallas, TX USA; 8 Department of Internal Medicine III, Group Artificial Intelligence in Cardiovascular Medicine, Heidelberg University Hospital, 69120 Heidelberg, Germany; 9 DZHK (German Centre for Cardiovascular Research), Heidelberg/Mannheim, Germany; 10 German Competence Network for Congenital Heart Defects, DZHK (German Centre for Cardiovascular Research), Berlin, Germany; 11 Department of Cardiothoracic, Transplantation and Vascular Surgery, Hannover Medical School, Hannover, Germany; 12 Department of Paediatric Cardiology and Congenital Heart Defects, German Heart Centre Munich, Munich, Germany; 13 Department of Pediatric Cardiology, Cleveland Clinic Children’s, Cleveland, OH USA; 14 Department for Paediatric Cardiology and Paediatric Intensive Care Medicine, University Children’s Hospital, Hannover Medical School, Hannover, Germany

**Keywords:** Tetralogy of Fallot, Biventricular shape, Magnetic resonance imaging, Biomarker

## Abstract

**Background:**

Maladaptive remodelling mechanisms occur in patients with repaired tetralogy of Fallot (rToF) resulting in a cycle of metabolic and structural changes. Biventricular shape analysis may indicate mechanisms associated with adverse events independent of pulmonary regurgitant volume index (PRVI). We aimed to determine novel remodelling patterns associated with adverse events in patients with rToF using shape and function analysis.

**Methods:**

Biventricular shape and function were studied in 192 patients with rToF (median time from TOF repair to baseline evaluation 13.5 years). Linear discriminant analysis (LDA) and principal component analysis (PCA) were used to identify shape differences between patients with and without adverse events. Adverse events included death, arrhythmias, and cardiac arrest with median follow-up of 10 years.

**Results:**

LDA and PCA showed that shape characteristics pertaining to adverse events included a more circular left ventricle (LV) (decreased eccentricity), dilated (increased sphericity) LV base, increased right ventricular (RV) apical sphericity, and decreased RV basal sphericity. Multivariate LDA showed that the optimal discriminative model included only RV apical ejection fraction and one PCA mode associated with a more circular and dilated LV base (AUC = 0.77). PRVI did not add value, and shape changes associated with increased PRVI were not predictive of adverse outcomes.

**Conclusion:**

Pathological remodelling patterns in patients with rToF are significantly associated with adverse events, independent of PRVI. Mechanisms related to incident events include LV basal dilation with a reduced RV apical ejection fraction.

**Supplementary Information:**

The online version contains supplementary material available at 10.1186/s12968-022-00877-x.

## Background

Morphological descriptions of the heart have been historically imaginative. “Le Cœur en Sabot” (or the boot-shaped heart) is one such description of the appearance of the heart on a plain radiograph of patients with tetralogy of Fallot (ToF) [1]. In patients with repaired ToF (rToF), surgical relief of the right ventricular (RV) outflow tract often leads to pulmonary valve disruption, which results in chronic regurgitation, severe RV dilation, and biventricular dysfunction. For the purposes of the current study, we use the term “adaptive remodelling” to describe heart shape and size changes which help satisfy the body’s oxygen demands while maintaining pressure within the physiological range [2]. Conversely, “maladaptive remodelling” refers to shape and size changes occurring due to an adverse cycle of metabolic and structural changes resulting in heart failure. Although the hemodynamic burden from chronic RV volume load may be tolerated without symptoms during childhood, evidence suggests that the incidence of arrhythmia, heart failure, and death increases substantially in adult life [3, 4]. The identification of adaptive vs maladaptive remodelling has been difficult due to a lack of quantitative tools for shape analysis. However, new shape analysis tools enable the re-evaluation of historical morphological descriptions.

Cardiovascular magnetic resonance (CMR) is considered the gold standard for assessing ventricular volumes, function, and flows in congenial heart disease [5]. Recently, biventricular atlases have enabled analysis of the RV and left ventricular (LV) shape changes simultaneously, enabling the detection of anomalous patterns of biventricular inter-relationships [6–8]. Although previous studies have focused on surrogate markers such as pulmonary regurgitation volume index (PRVI) to characterise adverse remodelling [8, 9], using surrogate endpoints as intermediate indicators of adverse outcomes may overlook important shape-related information associated with adverse remodelling but not captured by the surrogate marker itself. Here, we investigated relationships between shape and adverse events defined as death, life-threatening arrhythmias, and cardiac arrest in the German Competence Network for Congenital Heart Defects [10, 11].

We hypothesised that specific 3D biventricular shape features are associated with subsequent adverse events, and that these “maladaptive remodelling” scores are distinct from shape variations associated with PRVI. If so, these novel shape features may indicate specific maladaptive remodelling mechanisms leading to adverse outcomes, which can be distinguished from adaptive remodelling associated with PRVI.

## Methods

### Subjects

This study retrospectively re-analyzed anonymised images and associated data from a large cohort of adolescents and young adults with rToF recruited from a multicenter study of the German Competence Network for Congenital Heart Defects (study identifier: NCT00266188, title: Non-invasive Imaging and Exercise Tolerance Tests in Post-repair Tetralogy of Fallot—Intervention and Course in Patients Over 8 Years Old). Ethical approval was obtained and all participants gave informed consent. This rTOF dataset constitutes one of the most extensive compiled datasets of this pathology to date. The data was acquired at 14 different sites between 2005 and 2008 on 1.5T and 3T CMR scanners. The primary adverse outcome was defined as any cardiac arrest, ventricular tachycardia, or death until the latest outcome data collection point in 2019 with median follow-up time of ten years (Table 1). Further descriptions of clinical data can be found in [11, 12].

**Table 1:** Patient demographics

	All patients (n = 192)	Adverse outcomes (n = 16)	No adverse outcome (n = 176)
Gender [F (%)]	77 (40)	(5) 31	72 (41)
Height [cm]	163.3 ± 14.7	164.2 ± 12.3	163.2 ± 14.9
Weight [kg]	57.0 ± 18.9	55.0 ± 18.4	57.2 ± 19.0
BSA [m^2^]	1.59 ± 0.3	1.57 ± 0.3	1.6 ± 0.3
Median age at baseline exam [years](IQR)	15 (6.3)	16.5 (9.3)	15 (6)
Median age at ToF repair [years](IQR)	1 (3)	1 (1)	1 (3)
Median time from ToF repair to baseline exam [years] (IQR)	13.5 (5)	15 (6)	13 (5)
Median number of RCS before baseline exam [years]	0 (1)	1(1)	0 (1)*
PVR after baseline exam BE [# (%)]	27 (14)	2 (12) ^†^	25 (14)
Diagnosis [# (%)]			
ToF	161 (84)	14 (87)	147 (83)
Pulmonary atresia and VSD	27 (14)	2 (13)	25 (14)
DORV	4 (2)	0 (0)	4 (2)
Type of TOF repair [# (%)]			
Transannular patch	44 (23)	5 (31)	39 (23)
Transannular patch with MPA patch	40 (21)	3 (19)	37 (22)
No patch	62(32)	4 (24)	58 (33)
RV to PA conduit	23 (12)	2 (13)	21 (12)
Not defined	23 (12)	2 (13)	21 (12)
NYHA class [# (%)]			
I–II	188 (98)	192 (100)	172 (98)
III	4 (2)	0 (0)	4 (2)
Exercise parameters at baseline exam			
Peak heart rate	169 ± 20	160 ± 29	170 ± 19
Peak VO_2_ (mL VO_2_/kg/min)^‡^	31.8 ± 8.9	28.3 ± 10.8	32.1 ± 8.6

Normal distributed measurements are given in the format of mean ± std dev, p-values are computed using Welch's test. Non-normal distributed measurements are given in the format of median (IQR) and p-values are computed using Mood’s Median test. P-value annotation legend: *:0.01 < p <  = 0.05; ^†^One patient had PVR before baseline exam. Data were available for 188 patients

*BSA* body surface area, *PVR* pulmonary valve replacement, *DORV* Double outlet right ventricle, *MPA* main pulmonary artery, *NYHA* New York Heart Association, *PA* pulmonary artery, *PVR* pulmonic valve replacement, *RCS* redo corrective surgery, *RV* Right ventricular, *ToF *tetralogy of Fallot, *VSD* ventricular septal defect

### CMR imaging

CMR cine exams included multiple short-axis slices covering both RV and LV and one four chamber (4Ch) long-axis slice acquired during breath-hold. End-diastolic (ED) and end-systolic (ES) CMR images were segmented semi-automatically with a subsequent manual correction by a single reader. The LV endocardial, LV epicardial, and RV endocardial contours were defined using cvi42 software (Circle Cardiovascular Imaging, Calgary, Canada) according to the standards published by Society for Cardiovascular Magnetic Resonance [13]. Papillary muscles and trabeculae were included in the chamber volume for this analysis. Tricuspid and mitral valve hinge points and LV apex position were extracted from the 4Ch view. RV epicardial contours were not defined due to the poor reproducibility of manual delineation [14]. Pulmonary regurgitant volume was computed from phase contrast flow imaging across the main pulmonary artery, using flow measurements was performed using dedicated customized software capable of handling data of different vendors [15].

### Biventricular models

**Fig. 1:**
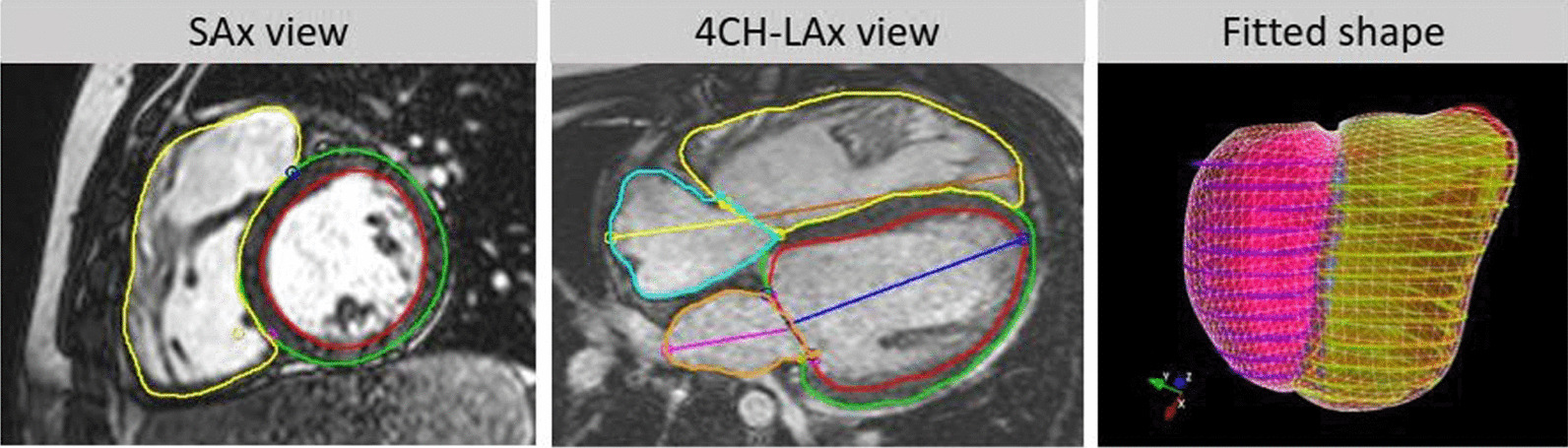
Patient-specific biventricular shape modelling. Left—short axis contours, middle—long axis contours, right: biventricular shape. Contours: green—left ventricular (LV) epicardium, red—LV endocardium, yellow—right ventricle (RV) endocardium, orange—left atrium, cyan—right atrium. Surfaces: (right) green—RV, purple—LV

A biventricular model template was automatically customised to the extracted contours using previously validated methods [6, 7]. Briefly, short axis slice positions were first corrected for breath-hold misregistration, and the template was then rigidly aligned and fit to the contours using a diffeomorphic non-rigid registration (see Fig. 1).

### Volume and mass

Global functional and volumetric parameters were computed from the patient-specific shape model, including ED and ES volumes (LVEDV, RVEDV, LVESV, RVESV), stroke volumes (LV SV, RV SV), LV mass (LVM) and ventricular ejection fraction (LVEF, RVEF).

**Fig. 2:**
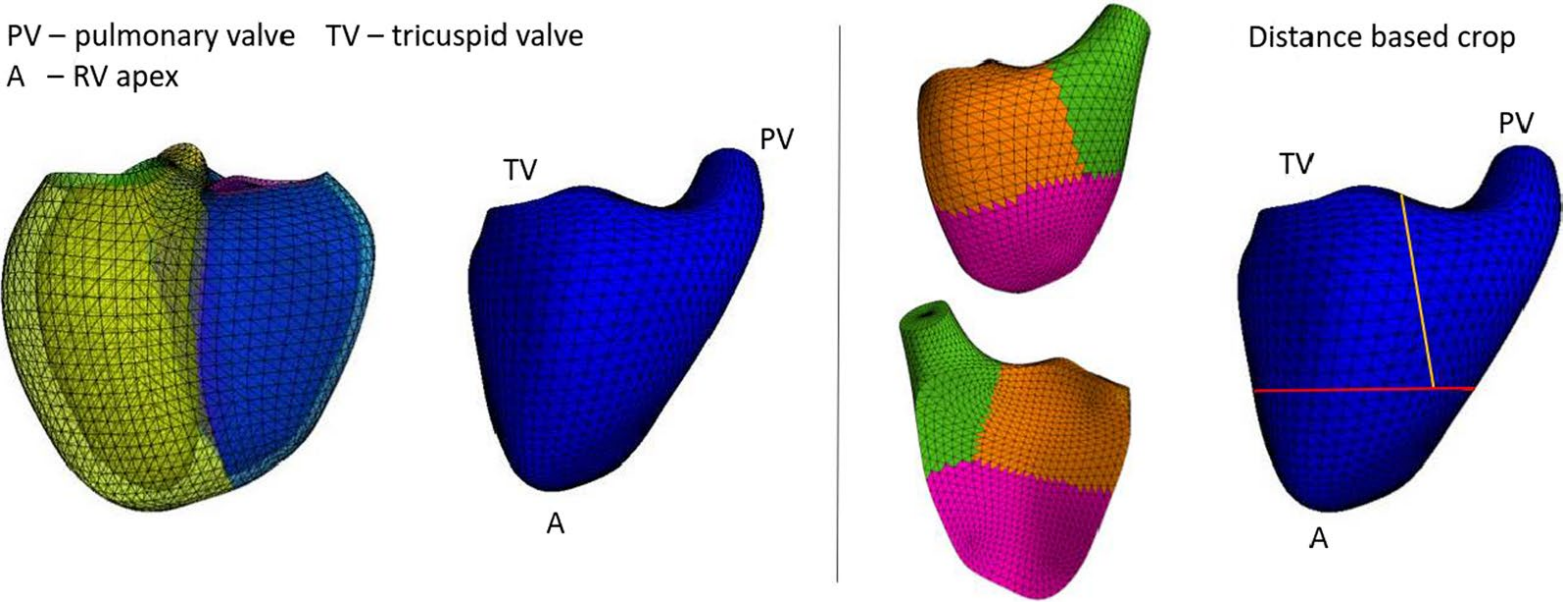
Volume parcellation. The right ventricle (RV) mesh is cropped according to the landmarks’ closest distance (PV—pulmonary valve, TV—tricuspid valve, A—RV apex). Cyan—inlet volume, green—outlet volume, and purple—apical volume

Besides global volume, regional volumes were also computed using methods similar to Bernardino et al. [16]. The total RV volume was parcellated into three different regions based on RV apex, pulmonary valve, and tricuspid valve centroids position (Fig. 2). The surface vertices were classified as inlet, outlet, and apical regions according to the closest landmark. For each region, partial volume, SV, and LVEF/RVEF were computed.

### Shape modes

Patient-specific biventricular geometries were used to build a statistical cardiac atlas characterising the shape variation in patients with ToF late after repair. Details of the procedure are given in [6, 8]. Briefly, a Procrustes algorithm was used to rigidly align the ED shapes to a reference coordinate system defined by the population mean shape. The same transformation was applied to the ES shape, thereby preserving their relative position and the surface deformation from ES to ED.

Principal component analysis (PCA) was performed to build a statistical shape model. Both geometry and motion were encoded by concatenation of the ED and ES shapes for each case. The statistical shape model included RV and LV endocardial and LV epicardial surfaces. However, for clarity, the figures displaying biventricular shape show the RV and LV endocardial surface only.

A PCA component, known as a shape mode (SM), reflects a specific shape variation pattern and encompasses attributes of size and both global and regional shapes. Any individual 3D shape can be characterised by a set of normalised scores (z-scores) specifying each SM’s contribution to the subject-specific geometry. For a robust and meaningful examination, only SMs explaining more than 1% of total variation were selected for further analysis. That resulted in 15 SMs, which in total explained 85% of the variation across the cohort.

### Maladaptive remodelling

Remodelling patterns associated with adverse events were identified using linear discriminant analysis (LDA) combined with an automatic feature selection algorithm according to the area under the curve (AUC) of the receiver operator characteristic, similar to the computational framework proposed by Varela et al. [17]. The shape features used in the LDA included conventional global and regional functional and volumetric measures together with the first 15 atlas-derived SMs. Prior to multivariable LDA, all clinical, demographic, or shape features were standardised and normalised. The ventricular volume and mass were indexed by body surface area (BSA) to mitigate the effects of body habitus.

Three separate preliminary LDAs were performed, each evaluated with stratified four-fold cross-validation (Additional file 1) designed to identify the shape features most associated with adverse outcomes: i) including only conventional measures (total ventricular volumes, EF, left ventricular mass (LVM) index (LVMI), QRS, and blood pressure); ii) regional functional and volumetric features derived from RV volume parcellation, and iii) including only the first 15 SMs. To avoid overfitting, the maximum number of predictors in each step was fixed to three parameters.

Finally, a composite LDA was performed by combining the predictors identified in the three preliminary analyses. The resulting LDA score was used as a new biomarker for risk quantification. The odds ratio (OR) with 95% CIs was computed to quantify the strength of the association with adverse outcomes. The cut-off value was computed by maximising the sensitivity and specificity in the ROC curves analysis.

### Remodelling associated with PRVI

A surrogate multivariable LDA model was developed to explore the role that PRVI may play in the association between shape and adverse events. This model included 158 cases for which pulmonary regurgitation data were available. The correlations between critical functional and shape predictors and PRVI were studied using univariable linear regression. Then, the shape mode best explaining PRVI was computed using multivariable regression.

### Calliper measurements

Atlas SMs describe both global and regional variation of ventricular shape, but their interpretation remains a challenging task. Calliper measurements have been used to quantify RV regional remodelling response in several pathophysiological conditions in imaging studies [18] and regional shape changes are important in rTOF patients [19]. In this work, multivariable regression was used to determine relationships between SM and calliper-based measures. The resulting associations were used to interpret the effect of the SM patterns.

**Fig. 3:**
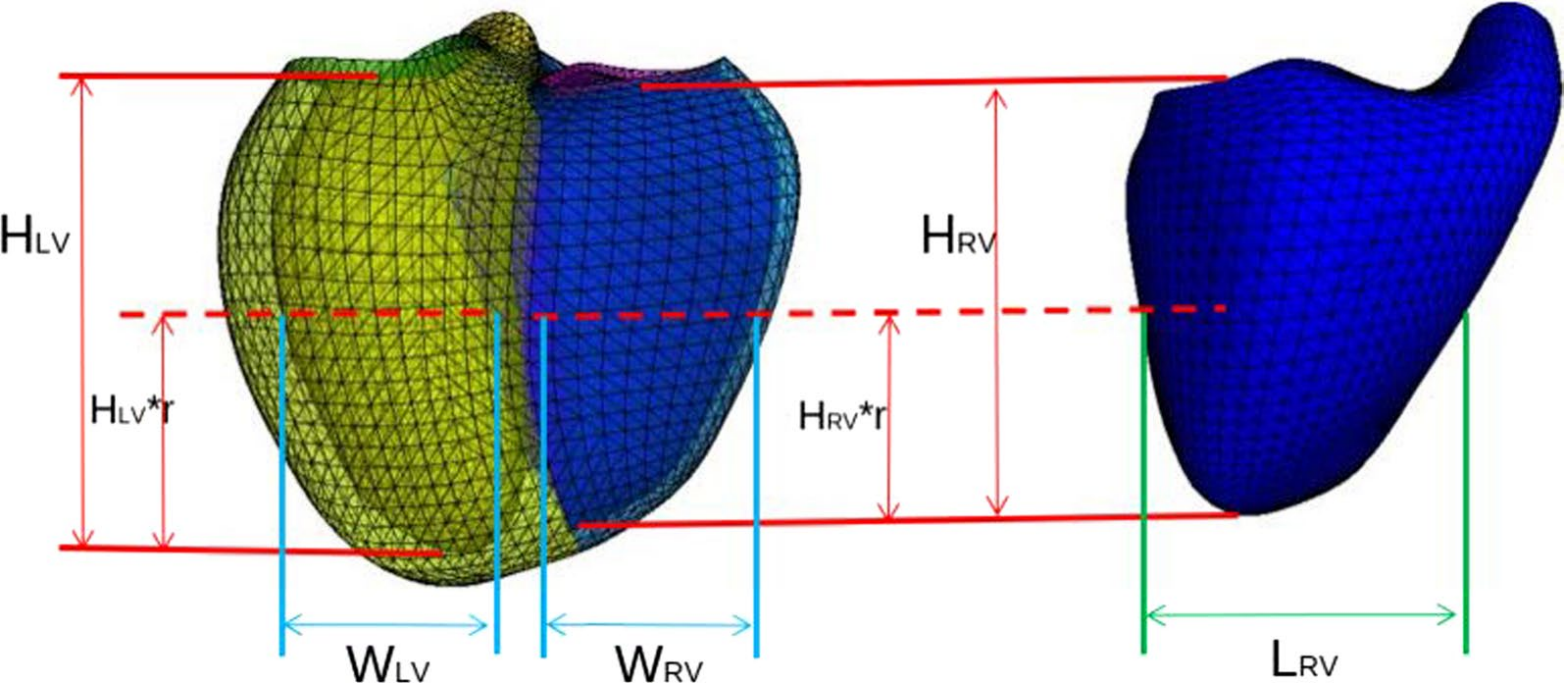
Definition of calliper distances. Yellow—left ventricle (LV). Blue—right ventricle (RV). HRV—the distance between tricuspid valve (TV) centroid and RV apex. HLV—the distance between the mitral valve (MV) centroid and RV apex. WRV—the distance between the RV free wall and the septum measured at mid RV height (r*HRV, r = ½) on the axis perpendicular to the septum. WLV—the distance between the LV endocardium and the septum measured at mid-LV height (r*HLV, r = ½) along the axis perpendicular to the septum. LRV—the distance between the two most distant points on the RV free wall at mid RV height (½*HLV) along the axis parallel to the septum

The calliper measurements were generalised for RV and LV chambers (see Fig. 3), as summarised in Additional file 1: Table S2. Ventricular sphericity (width divided by height) and eccentricity (anterior–posterior width divided by septal-lateral width) were computed for ED and ES using widths at 1/4 (apical), ½ (mid), and 3/4 (basal) of the ventricle height. Longitudinal shortening and apex displacement were computed for both RV and LV. Valve displacement was computed for mitral and tricuspid valves.

The multivariate regression was performed for each calliper-based measure independently using the standardised shape scores as independent variables. The magnitude of the regression coefficients defined the SM’s contribution to the calliper measurement. Finally, the shape scores were interpreted in terms of the effect on calliper distances using the respective regression coefficients to indicate the correlation strength.

### Statistics

Statistical analyses were performed using Python 3.6 using the Scikit-learn package (Python Software Foundation, Wilmington, Delaware, USA). A p value of 0.05 was considered significant.

## Results

### Subjects

Of 408 patients enrolled in the main study, 380 patients had CMR examinations, 280 of these had both long axis and short axis slices, and 192 had sufficient 3D information enabling biventricular shape analysis (a long axis view capturing mitral and tricuspid valves and the LV apex) (Additional file 1: Fig. S1). (Additional file 1: Table S1) shows that the characteristics of the 216 excluded cases had similar demographics to the 192 cases included, except for a higher number of pulmonic valve replacement (PVR) after the baseline exam and a higher number of redo surgeries prior to the CMR for the excluded group. Of the 192 patients examined, 16 subjects were identified with primary adverse outcomes (AO) and 176 with no adverse outcomes. Adverse outcomes included 4 deaths, 10 ventricular tachycardia and 2 aborted sudden cardiac death (cardiac arrest requiring defibrillation). Demographic and clinical data are summarised in Table 1. In the studied cohort, the median age at baseline evaluation was 15 years. Among the no adverse outcome group, 26 patients had a PVR in the following two years after baseline evaluation. In the adverse outcome group, one patient had PVR nine years earlier than baseline evaluation, and one patient had a PVR in the following year after baseline evaluation. Six per cent of the entire population had at least one redo surgery. The median number of redo surgical repairs in the adverse outcome group was significantly higher than in the no adverse outcome group.

Functional and volumetric variables are summarised in Table 2. RVEF and LVM was significantly reduced in the adverse outcome group. Partial volume and regional EF for the RV is shown in Table 3. Regional RVEF was significantly reduced in the apical and inlet regions. The total RV volumes were similar between groups; however, the adverse outcome group showed an increased ES inflow and apical regional volume.

**Table 2:** Volumetric and functional measurements

	All patients (n = 192)	Adverse outcome (n = 16)	No adverse outcome (n = 176)
DBP [mmHg]	64.1 ± 10.2	65.0 ± 11.3	64.0 ± 10.1
SBP [mmHg]	118.0 ± 14.4	117.4 ± 15.5	118.0 ± 14.3
QRS duration (ms)	147.2 ± 22.8	145.1 ± 30.6	147.4 ± 21.9
LVEDVI [ml/m^2^]	88.1 ± 18.2	101.3 ± 31.5	86.9 ± 15.8
LVESVI [ml/m^2^]	42.9 ± 14.8	53.5 ± 30.9	42.0 ± 12.1
LV SVI [ml/m^2^]	45.3 ± 9.1	47.7 ± 10.2	45.0 ± 8.9
LVEF [%]	52 ± 8	49 ± 10	52 ± 8
RVEDVI [ml/m^2^]	131.4 ± 33.3	138.4 ± 31.7	130.7 ± 33.3
RVESVI [ml/m^2^]	83.7 ± 25.7	94.7 ± 26.2	82.8 ± 25.5
RV SVI [ml/m^2^]	47.7 ± 15.2	43.9 ± 14.8	48.0 ± 15.2
RVEF [%]	37 ± 9	32 ± 9	37 ± 9*
LVMI [g/m^2^]	67.6 ± 12.2	77.0 ± 18.0	66.7 ± 11.0*
LV ED MVR [g/ml]	0.77 ± 0.12	0.78 ± 0.1	0.78 ± 0.12
LV ES MVR [g/ml]	1.40 ± 0.28	1.38 ± 0.33	1.41 ± 0.27

The values are given in the format of mean ± std. *DPB* diastolic blood pressure, *ED* end-diastole, *LVMI* left ventricular mass index, *EDVI* end-diastolic volume index, *EF* ejection fraction, *ES* end-systole, *ESVI* end-systole volume index. *LV* left ventricle, *MVR* mass to volume ratio, *RV* right ventricle, *SPB* systolic blood pressure, *SV* stroke volume. *p < 0.05 no adverse outcomes vs adverse outcomes

**Table 3:** Partial volumes and partial functional measurements

	All patients (n = 192)	Adverse outcome (n = 16)	No adverse outcome (n = 176)
RV ED IPVI [ml/m^2^]	59.3 ± 15.7	64.7 ± 13.0	58.7 ± 15.7
RV ES IPVI [ml/m^2^]	36.9 ± 12.2	42.9 ± 10.9	36.5 ± 12.6*
RV ED OPVI [ml/m^2^]	25.7 ± 10.0	25.0 ± 10.6	25.7 ± 9.9
RV ES OPVI [ml/m^2^]	18.2 ± 7.4	18.6 ± 8.6	18.1 ± 7.3
RV ED APVI [ml/m^2^]	46.3 ± 12.5	48.7 ± 11.3	46.1 ± 12.6
RV ES APVI [ml/m^2^]	28.6 ± 8.9	33.0 ± 8.4	28.3 ± 9.0*
RV IPSVI [ml/m^2^]	22.4 ± 7.2	21.8 ± 5.5	22.2 ± 7.8
RV OPSVI [ml/m^2^]	7.5 ± 5.0	6.4 ± 5.2	7.6 ± 5.1
RV APSVI [ml/m^2^]	17.7 ± 6.5	15.7 ± 6.3	17.8 ± 6.5
RV IPEF [%]	38 ± 9	34 ± 7	38 ± 9*
RV OPEF [%]	28 ± 15	24 ± 16	28 ± 16
RV APEF [%]	38 ± 10	32 ± 11	39 ± 10*

The values are presented in the format of mean ± s.d. *A* apical region, *I* Inflow, *O* outflow, *PEF* partial ejection fraction, *PSVI* partial stroke volume index, *PVI* partial volume index. *p < 0.05 no adverse outcomes vs adverse outcomes

### Maladaptive remodelling

In the first LDA model comparing conventional predictors (volumes and EF), the RVEF had the best ability to discriminate the patients with adverse outcomes (AUC = 0.72). The cross-validation feature selection algorithm could not find any combination of additional features improving the model predictive power without the risk of overfitting.

The second LDA model (using predictors derived from RV volume parcellation only) resulted in an equivalent discriminative power (AUC = 0.73) when using apical ejection fraction (RV APEF) as the unique predictor. Both models resulted in a negative correlation between the RVEF and the probability of having an adverse outcome (RVEF coeff = − 0.55, RV APEF coeff = − 0.69).

The third LDA model, which included only PCA SMs, the best discriminative power was obtained (AUC = 0.66) with a linear combination of 2 components: SM6 and SM8. Small but significant correlations were found between SM6 and RVEF and RV APEF (R^2^ = 0.097, p < 0.001 for RVEF and R^2^ = 0.179, p < 0.001 for RV APEF. However no significant correlation was found between SM8 and RV APEF or RVEF ( SM8: R^2^ = 0.004, p = 0.624 and R^2^ = 0.003 p = 0.582 respectively). Additional figures supporting these results are provided in Additional file 1: Fig. S2–S5.

**Fig. 4:**
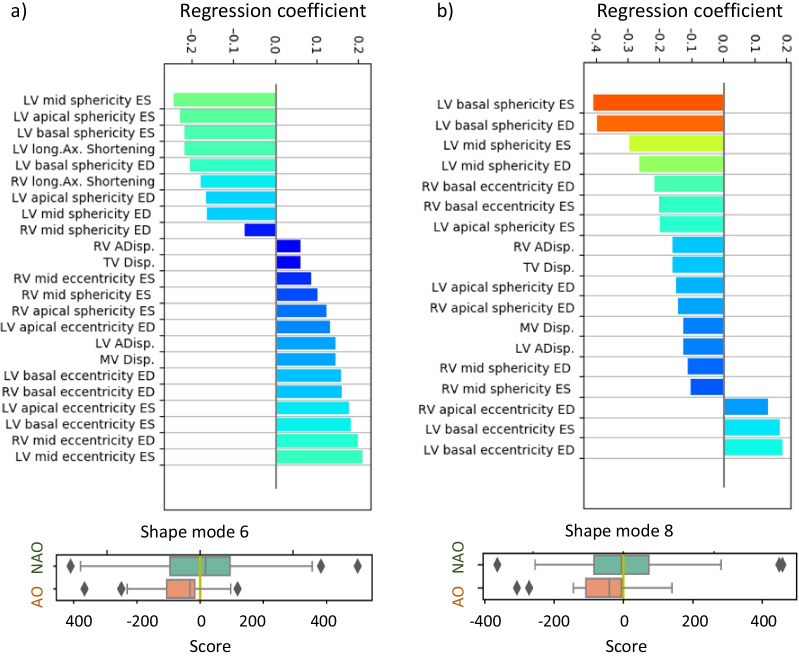
TOP: Association between geometrical features and the shape modes (SM): **a** SM6, **b** SM8. BOTTOM: Box-plots—score distribution of SM6 (**a**), SM8 (**b**) across the studied population for adverse outcomes (AO) and no adverse outcomes (NAO). The box denotes Q1 and Q3, whiskers Q1-1.5*IQR and Q3 + 1.5*IQR and diamonds are outliers. *ED* end-diastole, *ES* end-systole, *LV* left ventricle, *RV* right ventricle

**Fig. 5:**
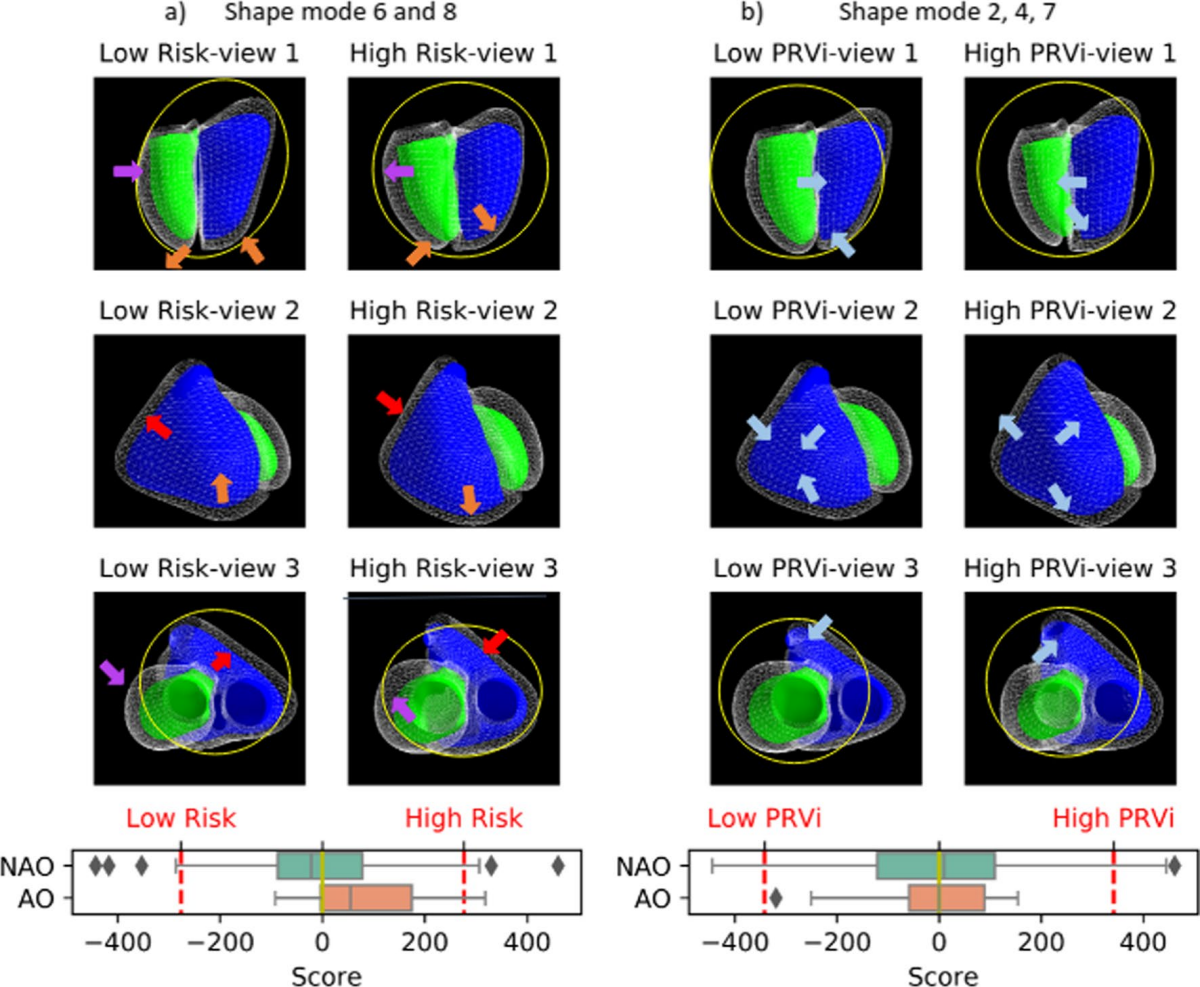
Shape pattern associated with adverse events **a** and pulmonary regurgitation (**b**). Low risk: shape associated with no adverse outcomes. High risk: shape associated with adverse outcomes. Low pulmonary regurgitation: shape associated with reduced pulmonary regurgitation. High P: shape associated with high pulmonary regurgitation. Green and blue surfaces show LV and RV (respectively) at ES. Wireframe: LV and RV at ED. Red arrow: RV basal remodelling; purple—LV basal remodelling; orange—RV apical remodelling and LV apical displacement; blue—RV apical dilatation. Yellow circles illustrate the contrast between circular shape versus elliptical shape. Box plots: **a** Distribution of the risk score for adverse outcomes and no adverse outcomes, b) distribution of PR score for adverse outcomes and no adverse outcomes. The box denotes Q1 and Q3, whiskers Q1-1.5*IQR and Q3 + 1.5*IQR and diamonds are outliers. Red lines: + 2std and −2std representing the score of the plotted shapes

**Fig. 6:**
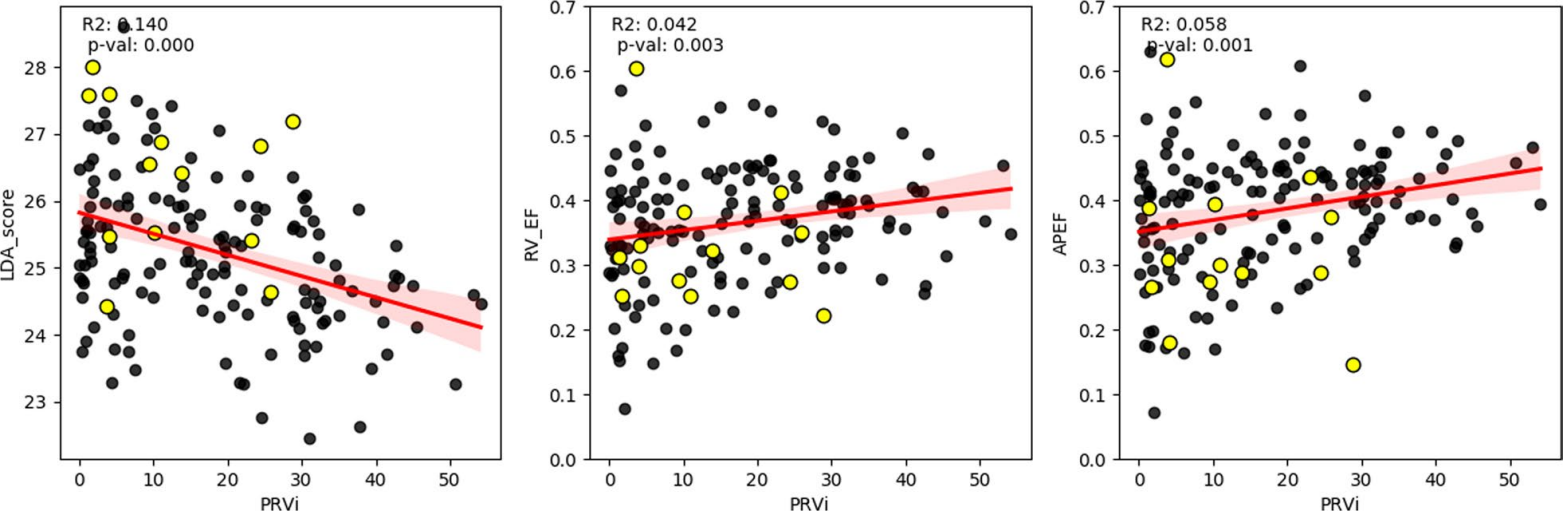
Association between pulmonary regurgitation and LDA risk score (SM6 + APEF), RVEF, and apical ejection fraction (APEF). Subjects with adverse events are marked in yellow circles

For ease of interpretation, the SMs most associated with adverse events were correlated with calliper measures (Fig. 4). Univariate analysis revealed no significant differences between adverse outcome and no adverse outcome groups. However, SM6 was found to be mostly correlated with LV ES shape and RV ED shape (Fig. 5). A negative SM6 (associated with higher risk of adverse outcomes) resulted in a more elliptical LV ES (decreased eccentricity) and a more annular RV base at ED (decreased mid and basal eccentricity—short axis). SM6 also significantly affected the mitral valve and LV apex position at the ES phase, resulting in a more annular LV at ES (increased sphericity) (Fig. 6). On the other hand, SM8 was mostly correlated with the biventricular mid and basal shape. In the adverse outcome group, the LV sphericity and RV eccentricity increased when compared to the no adverse outcome cohort. Additional material to visualize shape differences between the reference population and patients with adverse events can be found in Additional file 1.

The final multivariable LDA was built using the previously identified predictors: RVEF, RV APEF, and the two shape modes (SM6 and SM8). The optimal model included only RV APEF and SM6 and improved the discriminative performance from AUC = 0.73 to AUC = 0.77. A high LDA score was significantly associated with an increased odds ratio (OR = 2.06; 95% CI = 1.08 to 3.93). The cut-off value maximising sensitivity and specificity was equal to -0.18.

### Pulmonic regurgitation remodelling

A surrogate model using data from 158 subjects with available pulmonic regurgitation data were computed to investigate the impact on the model discriminative power (Additional file 1: Fig. S6). The LDA analysis showed that the PRVI had lower discriminative power than RV APEF (AUC = 0.60 versus AUC = 0.71) and had no added value when used as a predictor along with RV APEF and SM6 (AUC = 0.73 versus AUC = 0.72).

Further analysis was performed to investigate the PRVI’s relationship with the predictors of adverse events using linear regression (Fig. 6). There was a weak correlation (R^2^ = 0.14, p = 0.000) with the risk score, and no relation was found with either apical or total RVEF.

The shape pattern associated with PRVI was computed using LDA, and it resulted in a linear combination of 3 shape modes: SM7, SM2 and SM4 (R^2^ = 0.50) (Additional file 1: Fig. S7). The biventricular SMs associated with increased PRVI was compared to those identified with AO (Fig. 5). RV dilation was observed in both groups yet with different dilatation patterns. In patients with high PRVI, the RV dilatation was consistent over the RV volume (blue arrows). On the other hand, in the adverse outcome group, the RV dilatation was more prominent in the apical region, with RV basal constriction (red arrows). The combination of LV basal dilatation and RV base constriction led to an elliptical shape of the heart base in patients with adverse outcomes (yellow circle, view 3). Additional files 2 and 3: Videos S1 and S2 show a continuous animation of this remodelling.

## Discussion

This preliminary study investigated the relationships between adverse events and complex multi-dimensional biventricular shape features in rToF patients. Specific shape changes were found to be significantly associated with a subsequent composite endpoint of death, arrhythmia, or cardiac arrest. In particular, RV apical sphericity and RVEF were indicative of higher risk. We also identified “maladaptive remodelling” features using atlas-based shape scores associated with future adverse events. Furthermore, this study highlights new insights into the relationship between PRVI and adverse events. Our results suggest that remodelling associated with increased PRVI may not be in itself maladaptive. Differences were found in remodelling patterns between hearts ultimately associated with adverse events and hearts with increased PRVI. The old descriptor “Le Cœur en Sabot'', although originally used to describe a combination of adaptive and maladaptive remodelling, can also be used to describe the high-risk shape in rTOF. Here, shape changes show a more prominent toe of the boot corresponding to the dilated LV, revealed more in the anterior view in the high risk shape (Additional file 1).

Although identification of adaptive vs maladaptive remodelling is hampered by many factors, including tissue structure and mechanical factors, outcome-related shape change provides a new method for studying the mechanisms behind the development of heart failure. In particular, this study supports previous findings that traditional indices, such as ejection fraction (EF), mass, or blood-pool volume, do not fully utilise the rich information provided by the CMR data and may not be sufficient to allow early identification of patients at high risk [20]. Previous studies have also shown that ventricular shape features extracted from CMR images can provide additional information to quantify the unique, abnormal remodelling patterns occurring in adults with rToF [19, 21]. Initially, these studies relied on 2D regional shape indices extracted from CMR images. However, 2D measurements cannot capture the complex ventricular geometry, hence the more recent use of 3D models and atlas-based shape scores [6, 9, 22, 23]. Only a few studies have included the full 3D biventricular geometry [6–8], the main focus remaining on the RV or LV ventricular shape independently [16, 17, 19, 21]. A strong correlation between RV and LV dysfunction was found in patients with rToF [24], and LV systolic function was already associated with ventricular tachycardia and death in this population [25]. Recently, right atrial area and RV longitudinal strain were significantly associated with adverse events [11].

The shape features derived in this study can be automatically computed, using machine learning image analysis combined with an automated modelling pipeline, as demonstrated recently for the LV [26, 27]. Routine computation of shape scores will add to the spectrum of imaging biomarkers, including tissue characterization, fluid dynamics, and stress imaging in order to improve patient characterization and risk prediction. The advantage of shape features is that they can be interpreted in terms of mechanical remodelling mechanisms, particularly regarding interactions between the two ventricles. In conjunction with longitudinal imaging, changes in shape scores could indicate deterioration, or conversely the benefit of a particular treatment.

Regarding the LV shape, the remodelling pattern that predicted adverse events displayed basal dilation and a decreased eccentricity index (Fig. 4), consistent with previous studies [9]. Additionally, the maladaptive SM6 also captured a difference in LV apical displacement from ED to ES phase: in patients with adverse events, the LV apex moves further toward the RV (paradoxical septal motion). By moving toward the RV, the ventricular septum contributes to the RV SV to the detriment of LV SV, therefore changing the RV pumping physiology [28]. This specific behaviour was supported by a reduced RV APEF and may be a sign of failure or apical contractile function impairment. These results are consistent with a previous analysis of 2D function in the same cohort, in which right atrial area and RV longitudinal strain were found to be most predictive of adverse events [11]. RV and LV volumes obtained from the 3D model were different from those obtained directly from the contours [11] due to differences in the methodology of volume calculation, although trends were similar.

Visual inspection of the 3D remodelling patterns further highlights that the RV maladaptive remodelling consisted of an RV apical dilatation in the diaphragm direction, while the outflow and basal regions were more constricted. In the study by Bodhey et al. [29], the authors analysed three RV compartments’ functional characteristics (apex, inflow, outflow) between patients with rToF and healthy subjects. The results showed that the inlet and outlet volumes (unlike apical volume) were not substantially affected by the conditions and extent of RV loading. Our data suggest that the inflow and outflow regions tend to dilate in patients with rToF with increased PRVI creating a basal bulge that is therefore considered adaptive. Nevertheless, the basal bulge is less evident in patients with adverse events suggesting that adaptive remodelling is suppressed (Fig. 5).

It should be noted that, in patients with adverse outcomes, the heart base (RV and LV as a complex) remodels towards an elliptical display in contrast to the no adverse outcome group where the base is maintaining a more circular display (see Fig. 5, view 3). These observations may be a combined consequence of changes in external loads imposed by surrounding anatomical structures (e.g., a more relaxed or elastic diaphragm or a larger atrium taking over space in the pericardial sac) and in ventricle properties (relatively softer material properties in RV apical region or LV basal region). However, it remains uncertain which events are the cause and which ones are the effect in this specific global remodelling process.

Our study agrees with previous studies which did not find independent associations between pulmonary regurgitation and adverse outcomes. Although initial studies have shown an association between pulmonary regurgitation and the occurrence of adverse events [3, 30], later studies with more detailed analysis have identified a multitude of other risk factors not directly related to pulmonary regurgitation [31]. Several studies have suggested that an increased pulmonary regurgitation is associated with RV dilation and outlet bulging [21, 23]. Consequently, similar shape patterns were intuitively associated with adverse outcomes. In the INDICATOR study [4], which followed 873 rToF patients over ~ 2 years, RV hypertrophy, LV and RVEF, and atrial tachyarrhythmias were predictive of outcomes (death and ventricular tachycardia), but not pulmonary regurgitation fraction. In our study, PRVI did not add value to the RV APEF + SM6 risk score, and a weak correlation was found with pulmonary regurgitation and RV apical dysfunction. Furthermore, evidence of different remodelling patterns was found between adverse outcomes and PRVI. In patients with increased PRVI, RV volume dilated almost uniformly, taking over the major part of the pericardial sac. Consequently, the LV size and sphericity were reduced. On the contrary, in patients at risk of adverse events, the remodelling process resulted in a specific apicobasal volumetric redistribution (Fig. 5).

## Limitations

Our study has several limitations. Heart failure was not included as an endpoint, since it was not an endpoint in the main study. Although surrogate measures can be difficult to define, future studies should evaluate symptomatic outcomes. This relatively young cohort may have different characteristics to older cohorts with a greater proportion of transannular patch repairs. A limited number of patients may impact the classification models’ statistical significance. Only 16 subjects with sufficient imaging data for 3D modelling had adverse events; thus, factors such as the effect of PVR could not be analysed. We did not remove cases with subsequent valve replacement (26 no adverse outcomes, 1 adverse outcome) or the case with PVR prior to baseline evaluation (1 adverse outcome), to avoid selection bias. Future work should include the analysis of a larger population to confirm our findings and control for heterogeneous factors. A larger dataset would also include other variables in the model without predisposing to type 1 errors. Future large studies incorporating temporal analyses through machine learning [11] will be able to examine the relationships between shape features and other indices such as dyssynchrony, strain, and atrial morphometry.

Over half the full cohort did not have adequate 3D slices to enable construction of a shape model (Additional file 1: Fig. S1). Additional file 1: Table S1 shows excluded patients had similar demographics but a higher number of PVR’s after the baseline exam and a higher number of redo surgeries prior to the CMR. The main reason for missing or inadequate long axis slices was variation in CMR protocol with many studies not capturing a true 4Ch view. Cases were not rejected due to scan quality or patient status, but further study is needed to determine the relationships between PVR, shape changes and outcomes. Furthermore, the dataset included only one long axis view (4Ch), resulting in relatively sparse contours in the RV outflow tract region. In patients with rToF, dilatation may occur around the pulmonary valve position as a result of RV remodelling. If not captured by short axis contours, such geometrical patterns were partially unseen by the statistical atlas. Finally, RV epicardial contours were not defined in this study due to poor reproducibility [14], although RV hypertrophy was found to be a predictor of adverse outcome in INDICATOR [4]. New machine learning methods [11] may enable a more reproducible delineation of the RV epicardium.

## Conclusion

Adverse events in ToF are associated with changes in biventricular geometry, with increased LV basal sphericity in combination with increased RV apical sphericity. Maladaptive cardiac remodelling corresponds to specific changes in shape, rather than size, suggesting different disease mechanisms leading to adverse events from adaptive shape changes related to RV volume overload. A further assessment of shape and regional functional features in a larger cohort is needed to determine whether these measures independently predict adverse outcomes in patients with rToF.

## Supplementary Information

**Additional file 1. **Additional data.

**Additional file 2: Video S1.** Animations of shape variations associated with adverse outcomes.

**Additional file 3: Video S2.** Animations of shape variations associated with pulmonary regurgitation.

## Data Availability

Data are available on request from the authors and GCN.

## Abbreviations

4Ch: Four chamber
APEF: Apical ejection fraction
BSA: Body surface area
CMR: Cardiovascular magnetic resonance
ED: End-diastolic
EF: Ejection fraction
ES: End-systolic
LDA: Linear discriminant analysis
LV: Left ventricle/left ventricular
LVEDV: Left ventricular end-diastolic volume
LVEF: Left ventricular ejection fraction
LVESV: Left ventricular end-systolic volume
LVM: Left ventricular mass
LVMI: Left ventricular mass index
PCA: Principal component analysis
PRVI: Pulmonary regurgitant volume index
PVR: Pulmonic valve replacement
rTOF: Repaired tetralogy of Fallot
RV: Right ventricle/right ventricular
RVEDV: Right ventricular end-diastolic volume
RVEF: Right ventricular ejection fraction
RVESV: Right ventricular end-systolic volume
SM: Shape mode
SV: Stroke volume
TOF: Tetralogy of Fallot
