# Supplementary material for: Le Cœur en Sabot: shape associations with adverse events in repaired tetralogy of Fallot
Source: J Cardiovasc Magn Reson. 2022 Aug 4;24:46. doi: 10.1186/s12968-022-00877-x (PMC9351245; doi:10.1186/s12968-022-00877-x)
Supplement: Supplementary file 1 — Additional file 1. Additional data. [file 12968_2022_877_MOESM1_ESM.docx]

**Supplementary Data**

*Participants*

Figure S1 shows participants included at each stage. In order to ensure adequate 3D modelling, cases were excluded if there were not enough images, and also if the long axis image did not adequately cover the LV apex and the tricuspid and mitral valves. Table S1 shows a comparison between the study cohort and the cases excluded due to inadequate images. Selection bias was minimal, except for a higher number of PVR’s after the baseline exam and a higher number of redo surgeries prior to the MRI in the exclusion group.

*Linear Discriminant Analysis*

Pathological remodelling patterns associated with AO were identified using linear discriminant analysis (LDA) combined with an automatic feature selection algorithm. The computational framework used in this paper was similar to the one proposed by Varela et al. [14]. Stratified four-fold cross-validation was performed to control for model overfitting. Considering the highly unbalanced data (only 16 subjects identified with adverse outcomes), low-dimensional models were prioritised (up to three predictors only).

The feature selection algorithm started with selecting the best univariable model in terms of cross-validated AUC. At each iteration (k), a new feature was selected according to the resulting AUC when combined with the subset of features selected at the previous iteration (k-1). Finally, N models were selected with the number of independent variables varying from 1 to N. The AUC and resubstitution gap (GAP) were then used to identify which cut-off point provided the best combination of training and testing metrics. The cut-off point defined the optimal subset of features identified by the algorithm as independently associated with the clinical outcome. The analysis was performed in Python 3.6 using the Scikit-learn package.


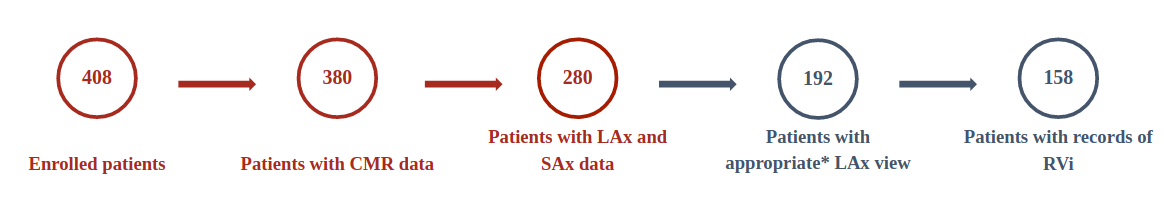


Figure S1: Patient numbers at each stage. *The long axis view was considered appropriate if the slice was capturing the mitral valve, the tricuspid valve and the LV apex at the same time. LAx - long axis view, SAx - short axis view, PR - pulmonary regurgitation.


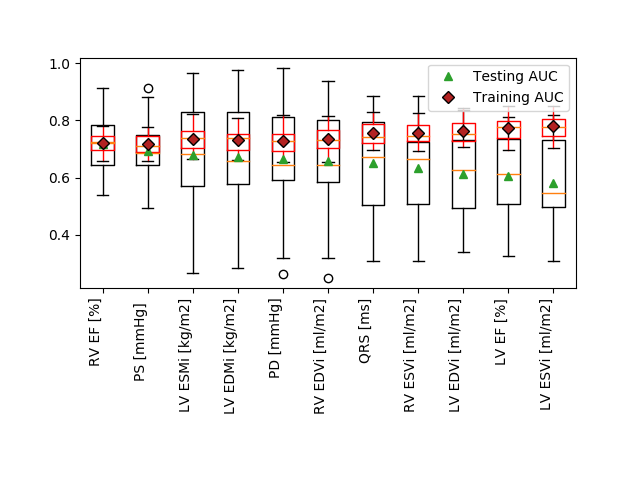


Figure S2: Result of LDA and feature selection relating the global functional and volumetric features to adverse events (first LDA model). LV: left ventricle, RV: right ventricle, EF: ejection fraction , EDVi: indexed end-diastolic volume, ESVi: indexed end-systolic volume, LV EDMi: LV indexed end-diastolic mass, LV ESMi: LV indexed end-systolic mass. PS: systolic blood pressure, PD: diastolic blood pressure.


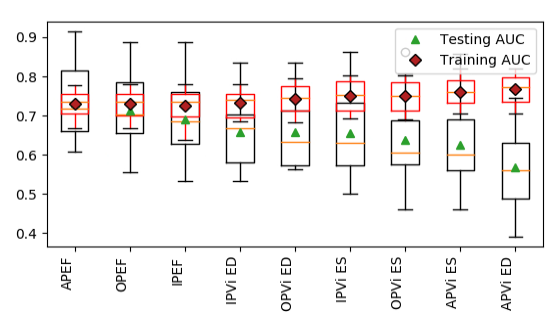


Figure S3: Result of LDA and feature selection relating regional functional and volumetric features to adverse events (second LDA model). APEF: RV apical ejection fraction, OPEF: RV outflow ejection fraction, RV IPEF: inflow ejection fraction, IPViED: indexed inflow volume at ED, OPViED: indexed outflow volume at ED, APViED: indexed apical volume at ED.


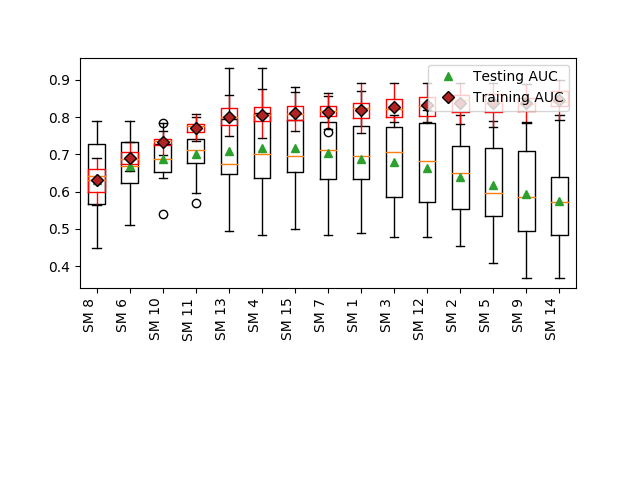


Figure S4: Result of LDA and feature selection relating the shape modes to adverse events (third LDA model). SM: shape mode.


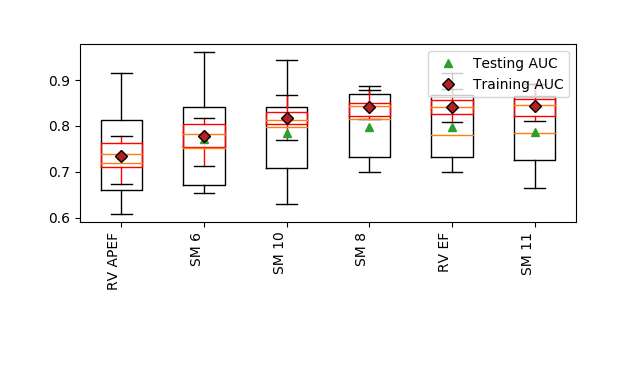


Figure S5: Result of LDA and feature selection relating the combined indicators to adverse events. SM: shape mode. AEF: apical ejection fraction.


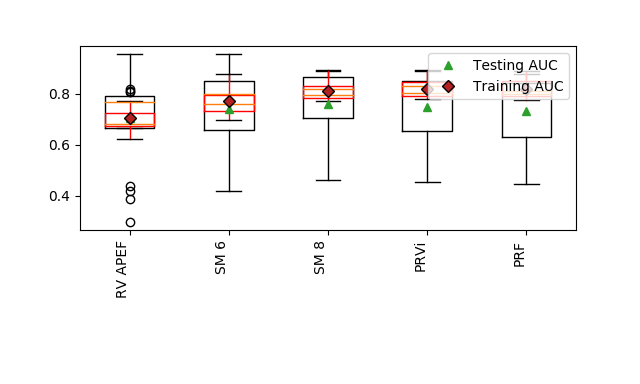


Figure S6: Result of LDA and feature selection relating the combined indicators to adverse events. The model is computed using 158 subjects for which the PRF was documented. SM: shape mode, RV: right ventricle, PRF: pulmonary regurgitant fraction, RV EF: right ventricular ejection fraction, AEF: apical ejection fraction.


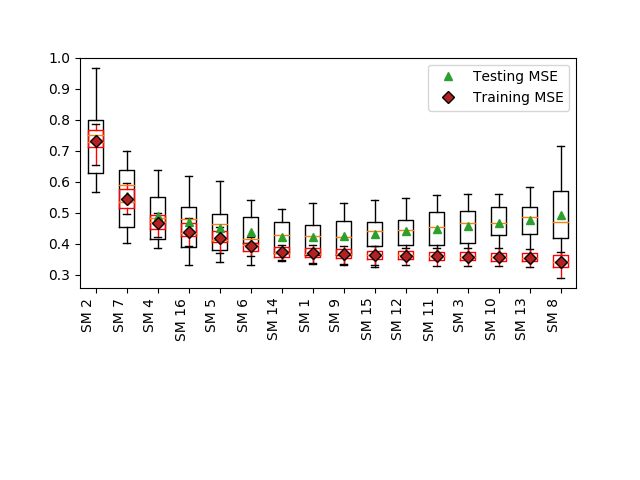


Figure S7: Result of LDA and feature selection relating shape modes to Regurgitant Volume index.

Table S1. Comparison between included patients (those with adequate MRI data) and excluded cases (see Figure S1). * p<0.001 Chi-squared test; $ p<0.001 Mann-Whitney U test. Normal distributed measurements are given in the format of mean ± std and p-values. Non-normal distributed measurements are given in the format of median (IQR). BE: baseline evaluation, BSA: body surface area, PVR: pulmonary valve replacement, DORV: Double outlet right ventricle, MPA: main pulmonary artery, RCS: redo corrective surgery.

|  | **Included patients (n=192)** | **Excluded Cases**  **(n=216)** |
| --- | --- | --- |
| **Sex [F # (%)]** | 77 (40) | 102 (47) |
| **Diagnosis [#( %)]** |  |  |
| **ToF** | 161 (84) | 193 (89) |
| **Pulmonary atresia** | 27 (14) | 17 (8) |
| **DORV** | 4 (2) | 6 (3) |
| **Type of TOF repair [#(%)]** |  |  |
| **Transannular patch** | 44 (23) | 43 (20) |
| **Transannular patch with MPA patch** | 40 (21) | 37 (17) |
| **No patch** | 85 (44) | 113 (53) |
| **Not defined** | 23 (12) | 23 (11) |
| **NYHA class [# (%)]** |  |  |
| **I-II** | 188 (98) | 211 (98) |
| **III** | 4 (2) | 5 (2) |
|  |  |  |
| **PVR after BE [# (%)]** | 27 (14) | 120 (56)* |
| **Median age at BE [years](IQR)** | 15 (6.25) | 16 (7) |
| **Median age at ToF repair [years](IQR)** | 1 (3) | 1 (3) |
| **Median time from ToF repair to BE [years] (IQR)** | 13.5 (5) | 14 (6) |
| **Median number of RCS before BE [years]** | 0 (1) | 0 (1)^$^ |
| **Height [cm]** | 163.3±14.7 | 162.1 ± 14.8 |
| **Weight [kg]** | 57.0±18.9 | 55.6 ± 17.5 |
| **BSA [m^2^]** | 1.59±0.33 | 1.57±0.31 |
| **Exercise parameters at BE** |  |  |
| **Peak heart rate** | 169±20 | 168±23 |
| **Peak VO2 (mL VO2/kg/min)**^‡^ | 31.78±8.9 | 30.8±9.1 |

Table S2: Definition of calliper-based measurements.

| **Measurement** | **Definition** |
| --- | --- |
| Sphericity | W / H |
| Eccentricity | L / W |
| Longitudinal shortening | (H ED - H ES) / L ED |
| Apex displacement | (AP ED - AP ES) / H |
| Valve displacement | (VP ED - VP ES)/ H |

AP = apex position; ED = end-diastolic; ES = end-systolic; H = distance between the valve centroid and apex; L = distance between the two most distant points of the endocardium on the axis parallel to septum; VP = valve position; W = distance between endocardium and septum on the axis perpendicular to the septum (see Figure 3).

Video S1.

Animations of shape variations associated with adverse outcomes shape_mode_AO.mp4

Video S2

Animations of shape variations associated with pulmonary regurgitation shape_mode_PR.mp4
