# Supplementary figures and images for: Le Cœur en Sabot: shape associations with adverse events in repaired tetralogy of Fallot
Source: J Cardiovasc Magn Reson. 2022 Aug 4;24:46. doi: 10.1186/s12968-022-00877-x (PMC9351245; doi:10.1186/s12968-022-00877-x)

## Slide 1
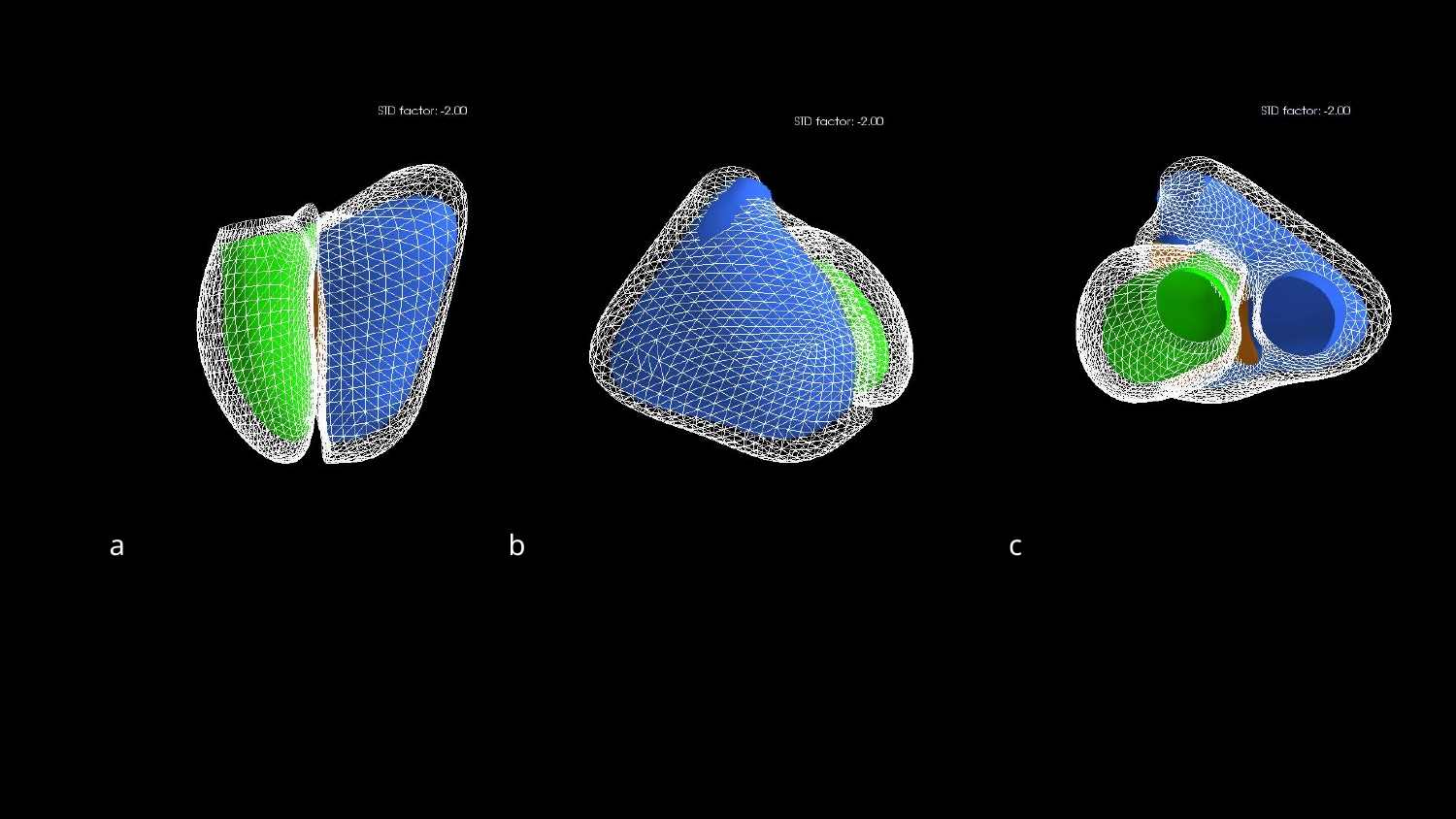

a
b
c

Supplement: Supplementary file 2 — Additional file 2: Video S1. Animations of shape variations associated with adverse outcomes. [file 12968_2022_877_MOESM2_ESM.pptx]

## Slide 1
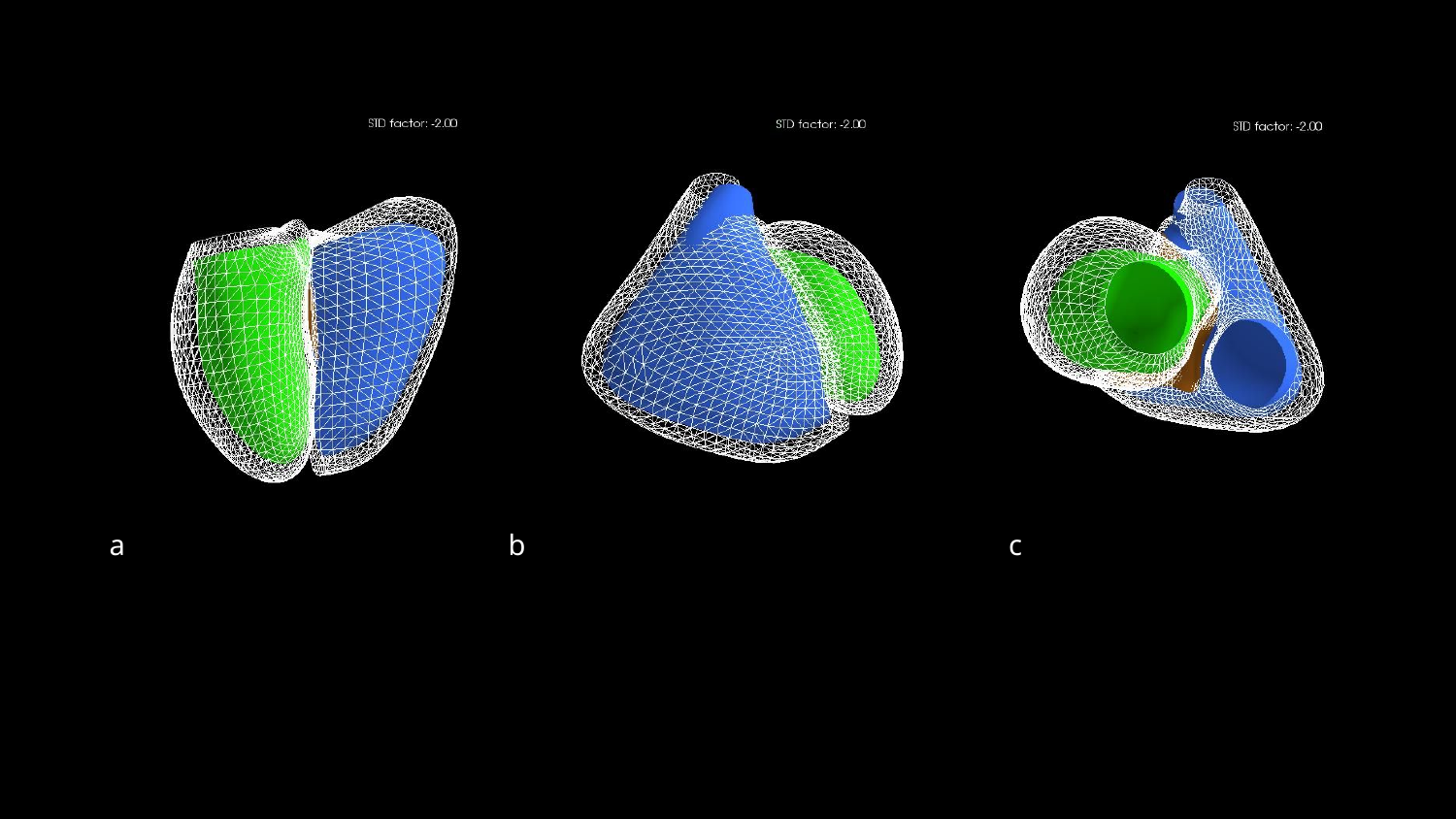

a
b
c

Supplement: Supplementary file 3 — Additional file 3: Video S2. Animations of shape variations associated with pulmonary regurgitation. [file 12968_2022_877_MOESM3_ESM.pptx]
